# Comparison of endoscopic versus microscopic transsphenoidal surgery in patients with pituitary adenomas: a propensity score matched study

**DOI:** 10.1097/JS9.0000000000002889

**Published:** 2025-06-24

**Authors:** Chengkai Zhang, Shunchang Ma, Dan Xiao, Luwen Zhang, Songbai Gui, Pinan Liu, Jingyao Geng, Huizhen Yang, Xuyang Zhao, Chuanbao Zhang, Xiudong Guan, Dainan Zhang, Liwei Zhang, Deling Li, Wang Jia

**Affiliations:** a Department of Neurosurgery, Beijing Tiantan Hospital, Capital Medical University, Beijing, China; b Department of Neurotomy, Beijing Neurosurgical Institute, Beijing, China; c China National Clinical Research Center for Neurological Diseases (NCRC-ND), Beijing, China; d School of Public Health, Capital Medical University, Beijing, China

**Keywords:** endoscopic, microscopic, pituitary adenoma, propensity score, surgery, transsphenoidal

## Abstract

**Background:**

The comparative efficacy of endoscopic versus microscopic transsphenoidal surgery for patients with pituitary adenoma (PA) remains controversial. Previous retrospective studies have often been limited by inconsistencies in baseline characteristics of patients, which may affect the validity of the comparisons. This study aimed to evaluate the trend in the proportion of transsphenoidal endoscopic and microscopic surgeries in China and compare outcomes in comparable patients.

**Materials and methods:**

The National Brain Tumor Registry of China (NBTRC) database was queried to extract PA patients (2011–2021) for trend analysis. Stratified sampling was performed, and a 1:1 propensity score matching (PSM) was used to balance the patients’ baseline characteristics. Postoperative outcomes, complications, and prognosis were compared.

**Results:**

Among the nationwide 17 012 PA patients, there was a gradual increase in the proportion of endoscopic surgeries (annual percent change, 11.89%). Among the 1863 stratified patients, those who underwent endoscopic surgery had a higher preoperative recurrence rate and higher Knosp and Hardy grades (*P* < 0.05). Endoscopic surgery showed a similar gross total resection (GTR) rate to microscopy (55.6% vs. 54.9%, *P* = 0.886) in the real-world cohort and a higher GTR rate (59.5% vs. 54.3%, *P* = 0.037) in the PSM cohort. After PSM, there was no significant difference in cerebrospinal fluid leak and secondary surgery (*P* > 0.05); endoscopic surgery showed more bleeding, longer surgical time, shorter hospital stay, and higher costs (all *P* < 0.001) compared to microscopic surgery. The risk of postoperative progression was similar between endoscopic and microscopic surgeries for comparable PA patients (*P* = 0.45).

**Conclusion:**

Endoscopic transsphenoidal surgery is increasingly adopted in China, demonstrating a higher GTR rate than microscopic transsphenoidal surgery in PA patients with similar characteristics, without increasing severe complication rates. The risk of postoperative progression was similar between the two techniques.

## Introduction

Pituitary adenoma (PA) is among the most common benign tumors of the central nervous system, accounting for approximately 15% of intracranial tumors[1]. Transsphenoidal resection of PA is a critical treatment for symptomatic patients^[2-4]^. Since the 1960s, microscopic transsphenoidal surgery has been a mainstream surgical treatment[5]. In 1992, Jankowski *et al* reported the first case of endoscopic transsphenoidal PA resection[6]. Since then, endoscopic surgery has gradually gained popularity^[7,8]^. Both endoscopic and microscopic surgeries share similar indications and procedures. Microscopic surgery is time-efficient and easy to operate, while endoscopic surgery provides broader visualization but requires a longer learning curve[9]. Despite the apparent trend of transitioning from microscopic to endoscopic techniques[8], the efficacy and safety of the two surgical methods remain controversial[9].

Lacking significant randomized clinical trials, numerous retrospective studies and meta-analyses have compared endoscopic and microscopic PA resection[10]. However, these retrospective studies commonly have limitations due to discrepancies in baseline characteristics between groups and cannot definitively demonstrate that one modality is superior to the other^[8,11–13]^. Only a few studies used propensity score matching (PSM) to balance discrepancies between groups. However, these studies were also limited by incomplete data, such as reporting only postoperative complications[8], and a vague definition of microscopic resection (both microscopic craniotomy and transsphenoidal surgery were classified as the microscopic group)^[7,9,14]^. Furthermore, these PSM studies reported contradictory results[9]. Therefore, we conducted a large-sample PSM study spanning 11 years to evaluate the two surgical modalities. This study aimed to estimate the trends, extent of resection, short-term complications, long-term follow-up, and health economic indicators of the two techniques.

## Method

### Study design and participants

This cohort study was performed based on the National Brain Tumor Registry of China (NBTRC)^[15–17]^, a hospital-based real-world brain tumor clinical database supported by the Chinese government. The NBTRC was approved by the ethics commit-tee of Beijing Tiantan Hospital, Capital Medical University (Beijing, China; KY 2019-124-02), and has been registered on the Chinese Clinical Trial Registry (ChiCTR1900021096). As of December 2021, a total of 50 hospitals from 28 provinces/ municipalities in China have participated in the NBTRC. All participating hospitals obtained approval from the central institutional review board. Informed consents were obtained from all patients, and the confidentiality and anonymity of medical information were fully guaranteed. Patients’ information was accessed through medical records, and Magnetic Resonance Imaging (MRI) images were obtained from the imaging system. This study followed the Strengthening the reporting of cohort, cross-sectional, and case-control studies in surgery (STROCSS) 2025 guidelines for observational cohort studies (Supplemental Digital Content 1, available at: http://links.lww.com/JS9/E489)[18].

**Figure 1.**
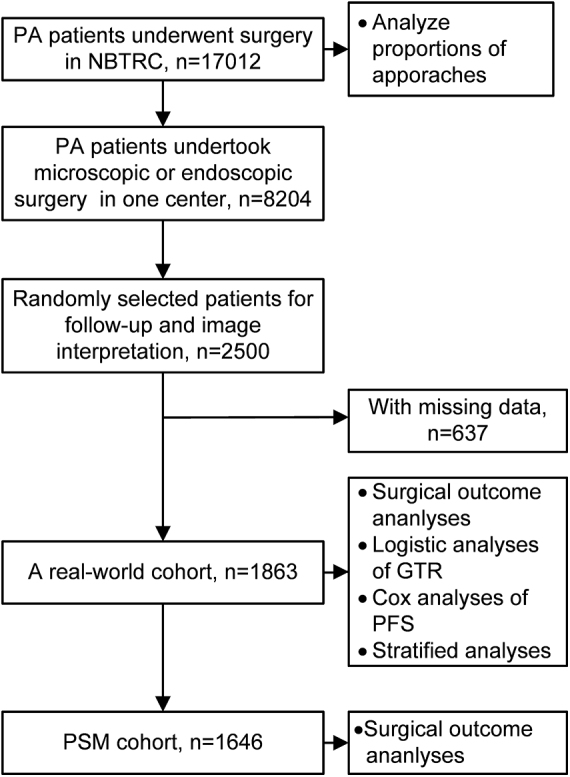
A flowchart describing the patients’ composition and analysis process.

As shown in Fig. 1, PA patients who underwent surgery from January 2011 to December 2021 in the NBTRC were included to analyze the changes in the proportion of approaches over the past years. To ensure data consistency by standardized protocols (identical equipment, surgical teams, and follow-up procedures), and to balance the feasibility of manpower allocation for time-consuming prospective follow-up and image interpretation, we randomly selected 2500 PA patients who underwent endoscopic or microscopic surgery at Beijing Tiantan Hospital, the largest neurosurgical center in China, for further detailed analyses. The single-center design inherently avoided inter-center variability, thereby strengthening internal validity through consistent application of protocols, and the randomization process minimized potential selection bias.

**HIGHLIGHTS**

- A nationwide multicenter study in China analyzed trends in endoscopic versus microscopic transsphenoidal surgery for pituitary adenoma (PA) patients over a decade, confirming the gradual adoption of endoscopic surgery.
- Propensity score matching was used to balance patients’ characteristics, enabling a more accurate comparison of the extent of resection, complications, and long-term prognosis between endoscopic and microscopic groups.
- For comparable PA patients, endoscopic surgery demonstrated a higher rate of gross total resection (GTR) compared to microscopic surgery, without increasing the risk of severe complications; and the risk of postoperative progression was similar.
- For tumors ≥25 mm, the advantage of endoscopic surgery in achieving GTR became more pronounced.

The inclusion criteria consisted of:

1. Adult patients who underwent microscopic or endoscopic transsphenoidal PA resection from January 2011 to December 2021 at Beijing Tiantan Hospital.
2. Postoperative pathological diagnosis of PA.
3. Complete preoperative and postoperative contrast MRI.
4. Minimal follow-up of 1 year.

The exclusion criteria included:

1. Unknown chief complaint or history of present illness; or unclear whether it is initial treatment or recurrence.
2. Lost of follow-up.
3. Hospitalized but did not undergo surgery.
4. Pituitary carcinomas with metastatic behavior.

### Data extraction

Demographic and clinical variables were captured in medical records, including age, gender, preoperative symptoms, preoperative recurrence or not, and disease course. Patients’ hormone types were classified through hormone tests. Tumor characteristics, including diameter, Knosp classification, and Hardy classification, were assessed through preoperative contrast MRI. Tumors were divided into three groups: microadenomas (<10 mm), macroadenomas (≥10 mm, <40 mm), and giant adenomas (≥40 mm). Primary outcomes included extent of resection (ETR) and progression-free survival (PFS). ETR was assessed through postoperative contrast MRI. Follow-up was conducted through postoperative MRI scans or telephone interviews. Progression was defined as recurrence after gross-total resection (GTR) or continued growth of tumor after incomplete resection. Secondary outcome measures, including surgical time, blood loss, and complications, were derived from operation records and postoperative medical records. Health economic variables, including hospitalization days and hospitalization costs, were extracted from medical records.

### Statistical analysis

Demographic and clinical characteristics of PA patients were described and compared between endoscopic and microscopic groups. Continuous variables that did not conform to a normal distribution were described using medians (25th percentile, 75th percentile) and compared using the Mann–Whitney U test. Ranked categorical variables were analyzed using the Mann–Whitney U test. Categorical variables were compared using the chi-squared test. Fisher’s exact test was also applied to compare rare events or subgroups with small sample sizes. PSM was performed at a 1:1 ratio to balance confounding variables (age, symptoms, hormone types, preoperative recurrent status, tumor size, Knosp, and Hardy levels) between endoscopic and microscopic groups. The propensity score was estimated by logistic regression model and matched using the nearest neighbor algorithm with a caliper value of 0.2. The Standardized Mean Difference (SMD) was calculated as the difference in means between two groups divided by the pooled standard deviation and was used to quantify the balance between groups. The SMD value <0.1 was considered indicative of a balanced distribution between the two groups. Surgical outcomes, surgical complications, and health economic indicators were compared between the two groups before and after PSM. Stratified analyses were performed in different characteristic subgroups to compare GTR rates between endoscopic and microscopic techniques using the propensity score-matched cohort. Furthermore, since the conventional grouping (microadenoma, macroadenoma, and giant adenoma) was somewhat generalized and offered limited guidance for surgical decisions. To address this issue, logistic regression with interaction terms was performed on the propensity score-matched cohort to evaluate whether the effect of the approaches on GTR rates varied across different tumor sizes. Grid search was used to identify the optimal tumor diameter cutoff where the ratio of GTR odds ratios (OR) between the two approaches was maximized for small and large tumors. Segmented logistic regression was performed to separately illustrate the tumor diameter-GTR probability curves in small and large tumors. Incremental cost-effectiveness ratio (ICER), defined as the ratio between the incremental cost and the incremental GTR rate of the two techniques, was applied for cost-effectiveness analyses. The Kaplan-Meier method was used to compare progression-free survival (PFS) between the two techniques. Univariable logistic regression was applied to analyze factors associated with GTR, and univariable cox regression was used to analyze risk factors associated with recurrence. The OR and 95% confidence interval (CI) for GTR of endoscopic versus microscopic surgery were compared across different subgroups. The study was performed using R (Version 4.4.0) program, with “pROC,” “rms,” ‘ggplot2ʹ, “survminer,” “survival,” and “MatchIt” packages. A *P* value <0.05 was considered statistically significant.

## Results

### Study population and demographics

**Figure 2.**
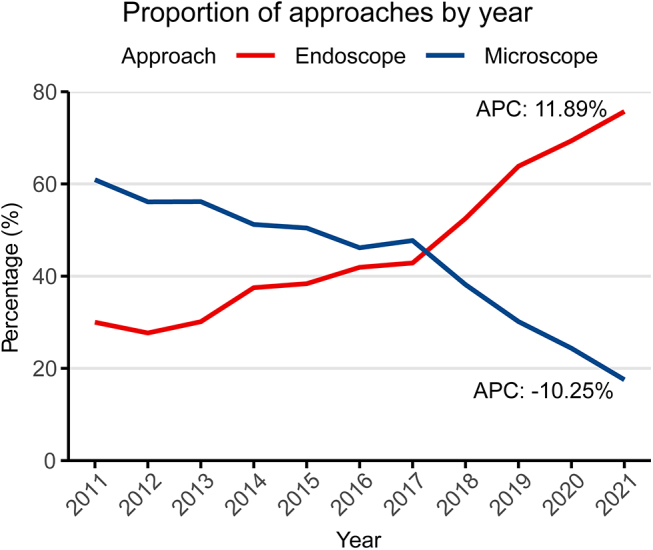
Changes in the proportion of endoscopic and microscopic transsphenoidal surgery from 2011 to 2021 (n = 17 012). Abbreviations: APC, annual percent change.

As shown in Fig. 1, 17 012 PA cases recorded in NBTRC were used to analyze changes in surgical proportions. The proportion of endoscopic surgery has gradually increased from 2011 to 2021, with an annual percent change (APC) of 11.89%. In contrast, the microscopic proportion has gradually decreased, with an APC of −10.25% (Fig. 2).

Subsequently, we randomly selected 2500 patients from the 8204 who underwent endoscopic or microscopic surgery at Beijing Tiantan Hospital for subsequent follow-up and approach comparison. After excluding patients who did not meet the criteria, 1,863 patients were included for further analyses (Fig. 1). The median follow-up was 34 months (IQR: 16–70 months). Patients in the endoscopic group had a higher proportion of preoperative recurrent tumors (15.7% vs 8.2%, *P* < 0.001). Meanwhile, endoscopic also showed higher Knosp and Hardy-sellar grades and more giant tumors (all *P* < 0.05). No differences were observed in age, gender, and disease course (all *P* > 0.05) (Table 1).

**Table 1:** Demographic and clinical characteristics of patients with pituitary adenoma who underwent endoscopic and microscopic surgery

Variable a	Endoscope	Microscope	Total	*P* value b
	(n = 1023)	(n = 840)	(n = 1863)	
Age (years)	48 (37, 57)	47 (38, 55)	47 (37, 56)	0.433
Gender				0.220
Female	543 (53.1%)	421 (50.1%)	964 (51.7%)	
Male	480 (46.9%)	419 (49.9%)	899 (48.3%)	
Preoperative recurrence	161 (15.7%)	69 (8.2%)	230 (12.3%)	<0.001
Symptomatic	922 (90.1%)	765 (91.1%)	1687 (90.6%)	0.539
Visual Decline	437 (42.7%)	407 (48.5%)	844 (45.3%)	0.015
Headache	262 (25.6%)	211 (25.1%)	473 (25.4%)	0.850
Hypopituitarism	74 (7.2%)	106 (12.6%)	180 (9.7%)	<0.001
Hormonal secretion-symptoms	322 (31.5%)	242 (28.8%)	564 (30.3%)	0.232
Disease course (month)	12 (3.0, 24)	12 (3.0, 24)	12 (3.0, 24)	0.226
Tumor size (mm)	26 (20, 34)	25 (20, 32)	26 (20, 33)	0.286
Size classification				0.021
Giant	140 (13.7%)	84 (10.0%)	224 (12.0%)	
Macro	872 (85.2%)	747 (88.9%)	1619 (86.9%)	
Micro	11 (1.1%)	9 (1.1%)	20 (1.1%)	
Knosp				<0.001
0	8 (0.8%)	10 (1.2%)	18 (1.0%)	
1	267 (26.1%)	266 (31.7%)	533 (28.6%)	
2	325 (31.8%)	319 (38.0%)	644 (34.6%)	
3	240 (23.5%)	150 (17.9%)	390 (20.9%)	
4	183 (17.9%)	95 (11.3%)	278 (14.9%)	
Hardy sellar				<0.001
0	1 (0.1%)	0 (0%)	1 (0.1%)	
1	89 (8.7%)	100 (11.9%)	189 (10.1%)	
2	298 (29.1%)	280 (33.3%)	578 (31.0%)	
3	361 (35.3%)	289 (34.4%)	650 (34.9%)	
4	274 (26.8%)	171 (20.4%)	445 (23.9%)	
Hardy suprasellar				0.072
A	265 (25.9%)	196 (23.3%)	461 (24.7%)	
B	231 (22.6%)	254 (30.2%)	485 (26.0%)	
C	423 (41.3%)	345 (41.1%)	768 (41.2%)	
D	79 (7.7%)	42 (5.0%)	121 (6.5%)	
E	12 (1.2%)	3 (0.4%)	15 (0.8%)	
DE	13 (1.3%)	0 (0%)	13 (0.7%)	
Hormone types				0.071
ACTH	27 (2.6%)	12 (1.4%)	39 (2.1%)	
CNFA	633 (61.9%)	546 (65.0%)	1179 (63.3%)	
GH	184 (18.0%)	120 (14.3%)	304 (16.3%)	
PRL	168 (16.4%)	152 (18.1%)	320 (17.2%)	
TSH	11 (1.1%)	10 (1.2%)	21 (1.1%)	
Year of surgery				<0. 001
2011–2015	188 (18.4%)	383 (45.6%)	571 (30.6%)	
2016–2018	212 (20.7%)	246 (29.3%)	458 (24.6%)	
2019–2021	623 (60.9%)	211 (25.1%)	834 (44.8%)	

^a^ The continuous variables in the table did not follow a normal distribution and were presented as median (Q1, Q3).

^b^ The *P* values were obtained by the Mann–Whitney U test for ordinal variables or non-normally distributed continuous variables, and the chi-squared test for categorical variables.

### Surgical outcomes, complications, and health economic indicators

In the real-world cohort, endoscopic and microscopic surgery showed similar extent of resection (ETR) (*P* = 0.886). The GTR rate was 55.6% in the endoscope group and 54.9% in the microscope (Table 2). Univariate logistic regression showed that PA patients with non-recurrence, small tumor diameter, short disease course, low Knosp and Hardy grades, hormone secretion symptoms, and no visual decline were more likely to achieve GTR. In contrast, endoscopic surgery did not show a statistically significant difference compared to microscopic surgery in terms of GTR (OR: 1.03, 95% CI: 0.86–1.24) (Supplemental Digital Content 2, Table 1, available at: http://links.lww.com/JS9/E490). At the same time, endoscopic surgery was associated with more cerebrospinal fluid (CSF) leaks, more bleeding, higher blood transfusion rates, and longer surgical time than microscopic surgery (*P* <0.05) (Table 2). Other surgical complications, including internal carotid artery (ICA) rupture and secondary surgery, did not show a difference between the two groups.

**Table 2:** Comparison of surgical outcomes, complications, and health economics indicators between endoscope and microscope before and after propensity score matching

	Before PSM			After PSM		
Variable a	Endoscope	Microscope	*P* value	Endoscope	Microscope	*P* value
	(n = 1023)	(n = 840)		(n = 823)	(n = 823)	
ETR			0.886			0.037
GTR	569 (55.6%)	461 (54.9%)		490 (59.5%)	447 (54.3%)	
STR	434 (42.4%)	370 (44.0%)		323 (39.2%)	367 (44.6%)	
PTR	20 (2.0%)	9 (1.1%)		10 (1.2%)	9 (1.1%)	
CSF leak	240 (23.5%)	158 (18.8%)	0.028	183 (22.2%)	156 (19.0%)	0.132
ICA rupture	1 (0.1%)	0 (0%)	1	0	0	1
Secondary surgery	3 (0.3%)	4 (0.5%)	0.794	1 (0.1%)	4 (0.5%)	0.374
Bleeding (ml)	100 (100, 200)	80 (50, 100)	<0.001	100 (100, 200)	80 (50, 100)	<0.001
Transfusion	40 (3.9%)	14 (1.7%)	0.006	25 (3.0%)	14 (1.7%)	0.105
Surgical time (min)	120 (90, 170)	84 (60, 100)	<0.001	120 (84, 160)	84 (60, 100)	<0.001
Hospital stay (days)	8.0 (6.0, 12)	8.4 (7.0, 11)	0.019	8.0 (6.0, 12)	8.4 (7.0, 11)	0.023
With complications^b^	10 (7.0, 13)	9.4 (7.4, 12)	0.524	10 (7.0, 13)	9.4 (7.4, 12)	0.567
Without complications	8.0 (6.0, 11)	8.0 (7.0, 11)	0.007	8.0 (6.0, 11)	8.0 (7.0, 11)	0.011
Cost (RMB)	36 116 (29 491, 43 476)	22 400 (16 226, 27 993)	<0.001	35 603 (28 630, 42 026)	22 423 (16 294, 28 028)	<0.001
With complications	41 399 (35 546, 51 326)	25 614 (21 075, 32 422)	<0.001	40 581 (34 515, 49 269)	25 614 (21 129, 32 542)	<0.001
Without complications	34 916 (27 785, 40 930)	22 062 (15 787, 26 782)	<0.001	34 129 (26 781, 40 459)	22 131 (15 893, 26 823)	<0.001

^a^ The continuous variables in the table did not follow a normal distribution and were presented as median (Q1, Q3).

^b^ Complications meant the occurrence of any of the following: CSF leak, ICA rupture, or secondary surgery.CSF, cerebrospinal fluid; ETR: extent of resection; GTR, gross total resection; ICA, internal carotid artery; PSM, propensity score matching; PTR, partial resection; STR, subtotal resection.

After PSM, endoscopic and microscopic groups showed no difference in confounding variables (all SMD < 0.1 and *P* > 0.05) (Supplemental Digital Content 2, Table 2, available at: http://links.lww.com/JS9/E490). In the PSM cohort, however, endoscopic surgery derived higher ETR (*P* = 0.037). The GTR rate of endoscopic surgery (59.5%) was higher than that of microscopic surgery (54.3%) (Table 2). The endoscopic group still exhibited more bleeding and longer surgical time than microscopic surgery, and the difference was statistically significant, while the surgical complications between the two groups were similar. The transfusion rate in endoscopic group (3.0%) was slightly higher than that in the microscopic group (1.7%), but the difference did not reach statistical significance (*P* = 0.105).

Hospital stay was significantly shorter in patients who underwent endoscopic surgery compared to microscopic surgery [8.0 (6.0, 12) days vs. 8.4 (7.0, 11) days, *P* = 0.019] (Table 2). This finding was consistent in the PSM cohort. For patients with complications, the length of hospital stay was similar between the endoscopic and microscopic groups. However, the endoscopic group demonstrated a shorter hospital stay than the microscopic group for patients without complications. The median cost of the entire cohort was 30 372 RMB. Endoscopic surgery cost more than microscopic surgery [36 116 (29 491, 43 476) vs. 22 400 (16 226, 27 993) RMB, *P* < 0.001], regardless of the presence of complications. In the PSM cohort, the ICER was 241 115 RMB per case of GTR, indicating that endoscopic surgery resulted in an additional cost of 241 115 RMB for each extra patient achieving GTR compared to microscopic surgery.

### Stratified analyses

**Figure 3.**
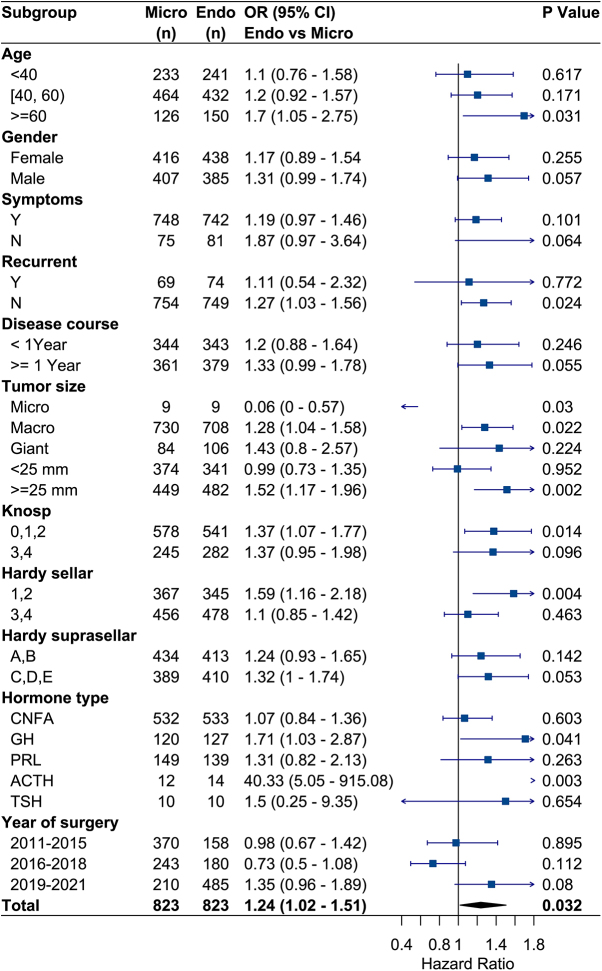
Comparison of gross total resection between endoscopic and microscopic surgery in different subgroups after propensity score matching. Abbreviations: Endo, endoscopic surgery; Micro, microscopic surgery.

Subgroup analyses were performed using the PSM cohort to compare the GTR of endoscopic and microscopic surgery in PA patients with different characteristics. As demonstrated in Fig. 3, endoscopic surgery showed a significantly higher probability of GTR than microscope in patients with ≥60 years (OR:1.7, 95% CI: 1.05–2.75), non-recurrence (OR: 1.27, 95% CI: 1.03–1.56), macroadenoma (OR: 1.28, 95% CI: 1.04–1.58), lower Knosp grade (OR: 1.37, 95% CI: 1.07–1.77), lower Hardy grade (OR: 1.59, 95% CI: 1.16–2.18), and GH adenomas (OR: 1.71, 95% CI: 1.03–2.87). Although endoscopic surgery seemed less likely to reach GTR for microadenomas, the small sample size (n = 9 for both endoscope and microscope) limited the ability to draw a meaningful conclusion. It is worth noting that the OR value of endoscope versus microscope for GTR had increased in recent years (2019–2021 subgroup, OR: 1.35, 95% CI: 0.96–1.89), although it did not reach statistical significance. This trend corresponded to the popularity of endoscopy in recent years. In other subgroups, there was no statistically significant difference in GTR rate between the two techniques (all *P* > 0.05).

In addition, the optical tumor diameter cutoff was identified as 25 mm. For tumors <25 mm, the probability of GTR was similar between endoscopic and microscopic groups (OR: 0.99, 95% CI: 0.73–1.35); for tumors ≥25 mm, endoscopic surgery showed a significantly higher GTR rate than microscopic surgery (OR: 1.52, 95% CI: 1.17–1.96) (Fig. 3). The ratio of the two OR values reached its peak, and the interaction between tumor size groups and surgical approaches was statistically significant (*P* = 0.038). Consistently, the segmented logistic regression also revealed that for tumors <25 mm, the GTR probabilities were similar between the two approaches. In contrast, for tumors ≥25 mm, the GTR probability was higher in the endoscopic group compared to the microscopic group (Supplemental Digital Content 2, Figure 1, available at: http://links.lww.com/JS9/E490).

### Progression-free survival

**Figure 4.**
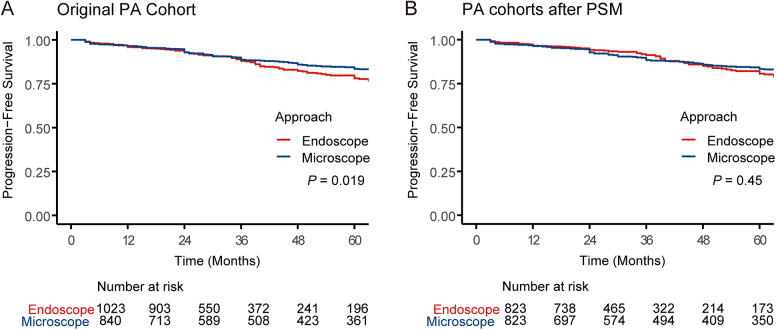
The progression-free survival of patients with pituitary adenomas after endoscopic and microscopic surgery in (A) the real-world cohort and (B) the propensity score-matched cohort.

In the real-world PA cohort, patients who underwent endoscopic surgery were more likely to progress than those who underwent microscopic surgery (*P* = 0.019) (Fig. 4A). However, after PSM, the risk of progression was similar in the endoscopic and microscopic groups (*P* = 0.45) (Fig. 4B).

Univariable Cox regression showed that PA patients with preoperative recurrence, large tumor size, and high Knosp and Hardy grades were more likely to progress (Supplemental Digital Content 2, Table 3, available at: http://links.lww.com/JS9/E490). On the other hand, PA patients with older age were less likely to suffer postoperative progression (HR: 0.99, 95% CI: 0.98–1.00).

## Discussion

### Study findings

Consistent with the global trend in the prevalence of neuro-endoscope^[7,8]^, this study showed a gradual increase in the proportion of endoscopic transsphenoidal surgery for PA in China. We found that endoscopic surgery was non-inferior to microscopy for GTR in a real-world cohort and had a higher GTR rate than microscopic surgery in similar patients without increased complications. The postoperative progression risk was similar between the two groups in patients with similar characteristics.

### Extent of resection

ETR, as the primary outcome of PA surgery, has been widely debated between endoscope and microscope. This PSM study showed that endoscopic surgery could reach better ETR. In the absence of randomized controlled trials, the majority of clinical studies comparing endoscopic and microscopic surgery were retrospective and prone to bias between the two groups. Despite most retrospective studies[19] and meta-analyses^[10,19–22]^ finding no statistical difference in GTR rates between the two groups, it was worth noting that several retrospective PSM studies suggested endoscopy was associated with higher GTR rates in similar patients^[9,23]^. These findings were consistent with our research. Additionally, only a few studies, such as Findlay’s research[9], found that microscopic surgery derived a higher GTR rate than endoscopy; however, the study also acknowledged that the endoscopic cases may be completed with less experienced neurosurgeons^[9,14]^. Therefore, there is reasonable evidence to suggest that endoscopic surgery is not inferior to microscopic transsphenoidal surgery in terms of ETR under comparable conditions.

Transsphenoidal microscopic pituitary surgery is a time-conserving technique with limited complications[5], and experienced neurosurgeons can achieve better outcomes. Microscope relies on three-dimensional visualization and is intuitive to operate. Endoscope provides extended visualization of deep structures; however, the two-dimensional visual field lacks depth perception, and the manipulation of instruments is more complex. Thus, endoscopy requires a longer learning curve[12]. Like most surgical procedures, the surgical technique is just one of several factors that influence outcomes, with surgical experience arguably being the most crucial^[24,25]^. Previous studies have found endoscopic transsphenoidal surgery skills could notably improve the initial several hundred cases[26], with further improvements observed even between 200 and 1000 cases[27]. The study of Hasan also found that a less experienced surgeon (100 cases) using an endoscopic technique could achieve similar outcomes to those of a very experienced surgeon (1800 cases) using a microscopic transsphenoidal technique when treating PA[28]. In the current study, the endoscopic group showed a slightly better GTR rate in later years. This improvement may mean that after sufficient time for promotion and training, endoscopic surgery could achieve better ETR.

The surgical device plays a role, but it is only a partial determinant of surgical outcomes. As mentioned above, logistic regression indicated that patients’ inherent characteristics, rather than surgical devices, were directly associated with the GTR in the real-world cohort. Moreover, choosing the appropriate strategy based on patients’ characteristics is also essential. For tumors ≥25 mm, the advantage of endoscopic surgery in achieving GTR became more pronounced. Consistently, a systematic review of Komotar also found that endoscopic surgery achieved a higher GTR rate than microscopic surgery for giant adenomas[29]. Endoscope overcame the limited visual field of the microscope[13], and demonstrated advantages in the resection of large tumors. Our results also showed that endoscopic surgery had a higher GTR rate than microscopic surgery in non-recurrent patients, while the GTR rate was similar between the two approaches in recurrent patients. Given the higher preoperative recurrence proportion in the endoscopic group, these findings may account for the differences in GTR outcomes between the real-world and PSM cohorts. Except for the well-known difficulty in resecting invasive PA[30], endoscopic surgery achieved relatively higher GTR rates than microscopic surgery for both invasive (high Knosp or Hardy grades) and non-invasive PA, and the differences reached statistical significance in the non-invasive group. The current research also found that endoscopic surgeries are more likely to achieve GTR than microscopic surgery in GH or ACTH adenomas, with ACTH adenomas typically being small. It may be attributed to the superior visualization of detailed structures provided by the endoscopic technique, which facilitated more precise and complete tumor resection. The findings of stratified analyses could provide valuable guidance for surgical decision-making in PA patients with different characteristics.

### Complication comparison

This research found that the endoscopic group had higher blood loss, which may potentially lead to higher transfusion rates. However, the overall perioperative transfusion rate in transsphenoidal PA surgery remained low (2.9% in the entire cohort) and was acceptable. Furthermore, endoscopic surgery did not increase the risk of other severe complications among comparable patients. Consistently, the majority of previous studies also found no difference in surgical complication rates between the two approaches^[20–22,31,32]^. We also found that the endoscopic group showed a higher rate of CSF leak in the real-world cohort, but no statistically significant difference was observed in the PSM cohort. It may be due to the higher Hardy grade in the endoscopic group, which indicated a more extensive invasion of sella, thereby increasing the likelihood of CSF leak. Furthermore, the management of CSF leaks was consistent between the two surgical techniques[33]. Therefore, the two techniques demonstrated equivalent safety.

### Health economics comparison

In addition to surgical outcomes and complications, health economic indicators are also important when choosing surgical modalities. Our study found that endoscopic surgery had longer surgical time, shorter hospital stays, and higher costs. These findings were also supported by previous studies^[19,34,35]^, while several other studies with conflicting conclusions may reflect institutional and health-economic differences^[36–38]^. A meta-analysis of Goudakos *et al* found that the length of hospital stays was significantly less in endoscopic groups[19]. Another systematic review also showed that the endoscopic group had shorter hospital stays than the microscope[34]. Endoscopic surgery enhanced postoperative recovery due to its reduced interference with the pituitary gland and minimal impact on the nasal mucosa^[9,39]^. Anthony’s study also showed that the costs associated with endoscopic surgery were significantly higher than microscopic surgery, especially for patients without complications[8]. The higher costs in the endoscopic group could be mainly attributed to increased equipment expenses, medical consumables application, and longer operative times[40]. Although endoscopic surgery was associated with higher costs, its potential benefits, such as improved GTR rate and enhanced postoperative recovery, may offset the increased financial burden. The higher GTR rate could reduce the need for postoperative interventions (e.g., radiotherapy, reoperations, and pharmacotherapy), and reduce the overall healthcare costs. Therefore, cost-effectiveness should be comprehensively considered when making surgical decisions.

### Progression-free survival

In terms of postoperative PFS, we found that PA patients who underwent endoscopic surgeries were more likely to progress than those who undertook microscopic surgery; however, the risk of progression was similar for patients with similar characteristics. This may be explained by the fact that the endoscopic group included more patients with preoperative recurrence and invasive PAs. There is a hypothesis that the endoscope became popular later than the microscope; consequently, recurrent PA patients who failed microscopic surgery were more inclined to choose endoscopic surgery. As mentioned above, patients’ initial characteristics were directly related to their likelihood of GTR. Therefore, patients who underwent endoscopic surgery, with a higher rate of preoperative recurrence, were more likely to experience progression after surgery. The similar risk of progression was consistent with previous research. A patient-level meta-analysis of 11 studies observed similar PFS between endoscopy and microscopy (HR 1.09, 95%CI 0.92–1.30, *P* = 0.301)[41]. Correspondingly, meta-analyses from Qiao[20] and Aydin[42] also found no difference in PFS between the two groups among patients with Cushing’s disease[20] and acromegaly[42]. Consequently, endoscopic and microscopic surgeries had similar long-term implications for PA patients. One possible explanation is that numerous factors could influence the postoperative recurrence of PA, with surgical equipment being a minor contributor.

We also found that older PA patients were less likely to experience postoperative progression. This could be attributed to the less aggressive tumor growth in older PA patients[43], or their limited life expectancy obscures the detection of tumor progression.

### Limitations

This research still has some limitations. Endocrine therapy and radiotherapy information were not included in the analysis, which may cause a bias in PFS. In addition, neurological function (e.g., visual acuity), other postoperative complications (e.g., postoperative hypopituitarism, and perioperative mortality), and detailed categories of hospitalization costs were not included in the analyses, which need further investigation. Moreover, recurrence patterns (e.g., anatomical location, and aggressiveness) were not recorded in this study, highlighting the need for future comparison between the two surgical techniques. Furthermore, although surgical trend analysis was supported by nationwide multicenter data, the comparison of the two approaches was based on a single center. Although this design ensured internal validity, factors such as surgeon experience and availability of medical resources may affect the applicability of the findings to other centers. Further studies are warranted to incorporate data from multiple centers, validating the robustness and enhancing the generalizability of our findings. Moreover, neurosurgeons had different surgical experience and tended to choose the specific approach they were most familiar with, which may lead to potential bias. Additionally, surgeon experience (years of surgeon experience or case volume) was not quantified in this study. Notably, the 1863 surgeries in this study were performed by over 20 surgeons with more than 10 years of experience, which might mitigate the potential bias caused by differences in surgeon experience.

## Conclusion

This study found the increasing adoption of endoscopic transsphenoidal surgery for PA in China. The findings suggest that endoscopic surgery is non-inferior to microscopy for achieving GTR, with a higher GTR rate observed in equally matched groups, without an increase in severe complications. Long-term follow-up showed no significant difference in PFS between the two groups when adjusted for patient characteristics.

## Data Availability

The data supporting the findings of this study are available from the corresponding authors upon reasonable request.

## Research registration unique identifying number (UIN)

Name of the registry: National Brain Tumor Registry of China.Unique identifying number or registration ID: Chinese Clinical Trial Registry, ChiCTR1900021096. Hyperlink to your specific registration (must be publicly accessible and will be checked): https://www.chictr.org.cn/showproj.html?proj=35120.
